# Assessing eukaryotic biodiversity in the Florida Keys National Marine Sanctuary through environmental DNA metabarcoding

**DOI:** 10.1002/ece3.4742

**Published:** 2019-01-15

**Authors:** Natalie A. Sawaya, Anni Djurhuus, Collin J. Closek, Megan Hepner, Emily Olesin, Lindsey Visser, Christopher Kelble, Katherine Hubbard, Mya Breitbart

**Affiliations:** ^1^ College of Marine Science University of South Florida Saint Petersburg Florida; ^2^ Stanford Center for Ocean Solutions Stanford University Stanford California; ^3^ Department of Civil and Environmental Engineering Stanford University Stanford California; ^4^ Florida Fish and Wildlife Conservation Commission‐Fish and Wildlife Research Institute Saint Petersburg Florida; ^5^ Rosenstiel School of Marine and Atmospheric Science University of Miami Miami Florida; ^6^ NOAA Atlantic Oceanographic and Meteorological Laboratory Miami Florida

**Keywords:** eDNA, 18S rRNA gene, cytochrome c oxidase I, monitoring

## Abstract

Environmental DNA (eDNA) is the DNA suspended in the environment (e.g., water column), which includes cells, gametes, and other material derived from but not limited to shedding of tissue, scales, mucus, and fecal matter. Amplifying and sequencing marker genes (i.e., metabarcoding) from eDNA can reveal the wide range of taxa present in an ecosystem through analysis of a single water sample. Metabarcoding of eDNA provides higher resolution data than visual surveys, aiding in assessments of ecosystem health. This study conducted eDNA metabarcoding of two molecular markers (cytochrome c oxidase I (COI) and 18S ribosomal RNA (rRNA) genes) to survey eukaryotic diversity across multiple trophic levels in surface water samples collected at three sites along the coral reef tract within the Florida Keys National Marine Sanctuary (FKNMS) during four research cruises in 2015. The 18S rRNA gene sequences recovered 785 genera while the COI gene sequences recovered 115 genera, with only 33 genera shared between the two datasets, emphasizing the complementarity of these marker genes. Community composition for both genetic markers clustered by month of sample collection, suggesting that temporal variation has a larger effect on biodiversity than spatial variability in the FKNMS surface waters. Sequences from both marker genes were dominated by copepods, but each marker recovered distinct phytoplankton groups, with 18S rRNA gene sequences dominated by dinoflagellates and COI sequences dominated by coccolithophores. Although eDNA samples were collected from surface waters, many benthic species such as sponges, crustaceans, and corals were identified. These results show the utility of eDNA metabarcoding for cataloging biodiversity to establish an ecosystem baseline against which future samples can be compared in order to monitor community changes.

## 1 INTRODUCTION

Coastal marine habitats are diverse and essential ecosystems that support many economically important industries including fisheries, tourism, and pharmaceuticals (Barbier et al., 2011; Moberg & Folke, 1999). Coastal ecosystems are facing unprecedented global threats ranging from climate change to habitat destruction (Kuffner, Lidz, Hudson, & Anderson, 2015). Defining the status of these ecosystems can be very difficult as different observational tools and techniques are needed to assess the function of each unique community of organisms, and metrics of health are highly dependent upon what humans value from the ecosystem (Lackey, 2001). With the awareness that each habitat functions differently, it is crucial to define a baseline for each ecosystem against which to compare future change.

The Florida Keys National Marine Sanctuary (FKNMS) covers over 9,500 km^2^ and is inhabited by >6,000 different marine species from diverse habitats such as seagrass beds, coral reefs, and mangroves (Suman, Shivlani, & Walter Milon, 1999). United States national marine sanctuaries were established to protect critical marine habitats; therefore, effective monitoring and maintenance of these sanctuaries is vital for assessing ecosystem condition. The current lack of a comprehensive biodiversity baseline for these sanctuaries makes it difficult to assess whether changes in the ecosystem result from natural fluctuations or are anthropogenically induced. Enhanced monitoring of marine sanctuaries allows for early detection of changes in key indicators to enable proactive management strategies, rather than relying upon reactionary responses (Port et al., 2016). Traditional visual surveys of marine habitats are time and labor‐intensive, often focusing on a few select taxa instead of observing the ecosystem as a whole. Additionally, traditional sampling tools are often not sufficient to detect all species and the temporal and spatial resolution is limited, emphasizing the need for new sampling techniques (Ardura et al., 2015). Assessment of marine protected areas such as the FKNMS would therefore benefit from an observation system that requires less sampling effort and simultaneously yields species information across multiple trophic levels.

Researchers have used metabarcoding (the amplification and sequencing of marker genes) of single‐celled organisms in water samples for over thirty years to describe the diversity and composition of environmental microbial and phytoplankton communities (Hugenholtz, Goebel, & Pace, 1998; Pace, 1997; Pace, Stahl, Lane, & Olsen, 1986; Pedro, Di, Massana, Marina, & De, 2001). These methods have recently expanded to encompass multicellular organisms by taking advantage of the fact that all organisms leave traces of their genetic material in the environment as environmental DNA (eDNA) through shedding and depositing waste (Deagle, Clarke, Kitchener, Polanowski, & Davidson, 2018; Taberlet, Coissac, Hajibabaei, & Rieseberg, 2012). Since the majority of eDNA is found in the 1–10 µm size fraction, a 0.22 µm filter effectively captures both single‐celled organisms and particulate organic matter left behind by multicellular individuals (Sassoubre, Yamahara, Gardner, Block, & Boehm, 2016; Turner et al., 2014). Therefore, metabarcoding of eDNA captured on a 0.22 µm filter from seawater enables high resolution examination of ecosystem biodiversity across multiple trophic levels (Biggs et al., 2015; Djurhuus et al., 2017; Jane et al., 2015; Kelly et al., 2017; Port et al., 2016; Stat et al., 2017). Additionally, since DNA degradation in the water column occurs within a few days to weeks, species recovered with eDNA are expected to have recently been present near the site of sample collection (Andruszkiewicz, Sassoubre, & Boehm, 2017; Thomsen et al., 2012)

As a part of the Marine Biodiversity Observation Network (MBON), which aims to monitor biodiversity across multiple trophic levels, we are testing the applicability of eDNA metabarcoding to examine eukaryotic communities by using routinely monitored and tightly regulated marine sanctuaries as sentinel sites that will act as indicators for the status of nearby marine ecosystems. Here, we compare the eukaryotic species identified through metabarcoding of two genetic markers, the 18S ribosomal RNA (rRNA) and cytochrome c oxidase I (COI) genes, from eDNA collected from the surface water at three coral reef sites in the FKNMS during four months (April, June, September, and November) in 2015.

## 2 MATERIALS AND METHODS

### 2.1 Sample collection

**Figure 1:**
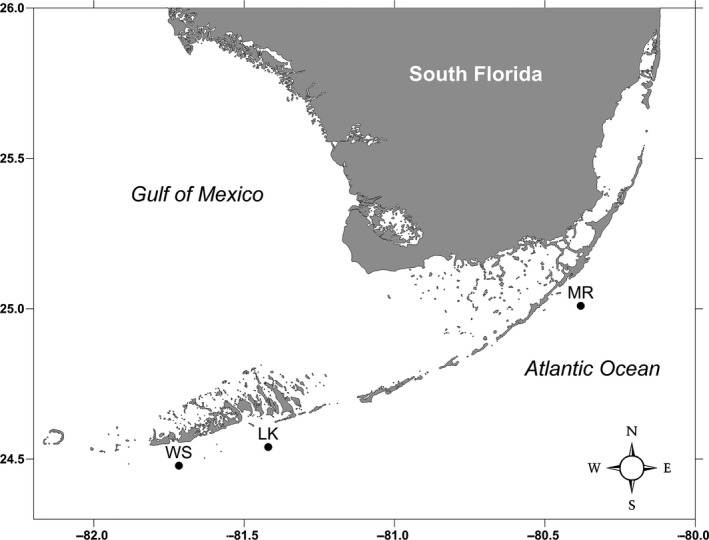
Map of South Florida depicting the three collection sites‐ Molasses Reef (MR) 25°00'36.0"N 80°22'48.0"W, Looe Key (LK) 24°32'18.0"N 81°24'48.0"W, and Western Sambo (WS) 24°26'40.8"N 81°43'01.2"W

Sampling was conducted in the FKNMS, at three sites along the reef tract: Molasses Reef (MR) 25°00'36.0"N 80°22'48.0"W, Looe Key (LK) 24°32'18.0"N 81°24'48.0"W, and Western Sambo (WS) 24°26'40.8"N 81°43'01.2"W (Figure 1). The total reef tract sampled was 149.25 km, MR and LK are 116.8 km from one another and WS is 32.45 km from LK. At each site, a rosette of Niskin bottles was submerged underwater and triplicate one liter surface seawater samples (one replicate from each Niskin bottle) were collected aboard the *R/V Walton Smith* throughout 2015 on April 13–14th, June 1st–2nd, Sept 21st−22nd, and November 16–17th. Onboard, the water was immediately filtered onto 0.22 µm polyvinylidene difluoride (PVDF) membrane Sterivex filters (Millipore). MilliQ water was also filtered onto a 0.22 µm PVDF Sterivex filter alongside field samples to serve as a filtration control. The filters were frozen in liquid nitrogen on the ship and stored in a −80˚C freezer until DNA extraction.

### 2.2 DNA extraction

Sterivex filters were opened with autoclaved pliers and the filters were removed from the cartridge using a sterile razor blade. Pliers were sterilized with DNA Away (Thermo Fisher Scientific) and a new razor blade was used for each filter. DNA was then extracted from the filters with the Qiagen DNeasy Blood and Tissue Kit (Qiagen) using the modified protocol described by Djurhuus et al. (2017) that includes a bead‐beating step for mechanical lysis of cells. Briefly, 1 g of 0.5 mm and 1 g of 0.1 mm glass beads (BioSpec Products) were added with 900 µl of ATL Buffer (Qiagen) to a 2 ml centrifuge tube containing the PVDF filter. The beads were sterilized by pre‐combustion at 500˚C for 3 hr before use. Bead‐beating was performed on a vortex with a bead‐beater adapter for 45 s at maximum speed, followed by a 30‐min incubation at 56 ˚C and another round of bead‐beating. Subsequently, 100 µl of Proteinase K (2 mg/L final concentration) were added, followed by 10 s of vortexing and a 2 hr shaking incubation at 56 ˚C. Samples were then vortexed for 15 s and centrifuged for 1 min (4,000 *g*), after which 650 µl of bead‐free supernatant was transferred into a new 2 ml tube. After these steps the DNeasy Blood and Tissue Kit (Qiagen) protocol was executed with the following modifications: 650 µl AL Buffer, 650 µl ethanol, and final elution steps of 2 × 50 µl AE Buffer. An extraction blank (no sample template) and the filtration control were processed alongside environmental samples.

### 2.3 PCR and library preparation

Primer sets targeting the 18S rRNA and COI genes were used to amplify each of the DNA extracts. The primer sequences for the 18S rRNA gene were: 1391F, 5′ GTACACACCGCCCGTC 3′, and EukBr, 5′ TGATCCTTCTGCAGGTTCACCTAC 3′ (Amaral‐Zettler, McCliment, Ducklow, & Huse, 2009). The primer sequences for the COI gene were: mlCOIintF, 5′ GGWACWGGWTGAACWGTWTAYCCYCC 3’ and HCO2198, 5’ TAAACTTCAGGGTGACCAAAAAATCA 3’ (Folmer, Black, Hoeh, & Lutz, 1994; Leray, Agudelo, Mills, & Meyer, 2013). Triplicate 25 μl PCR reactions were run using 12‐basepair Golay barcoded reverse primers (Amaral‐Zettler et al., 2009). Each reaction contained 1 μl of 1:10 diluted DNA extracts for the template, 10 μl Amplitaq Gold mastermix (Thermo Fisher Scientific), 1 μl each of forward and reverse primers (5 μM), and 4 μl of the mammalian blocking primer (10 μM) for 18S rRNA only (Amaral‐Zettler et al., 2018; Vestheim & Jarman, 2008). Triplicate PCR blanks (no template added) were run in conjunction with the samples following the same protocol.

18S rRNA cycling parameters were 94°C for 3 min, followed by 35 cycles of 94°C for 45 s, 65°C for 15 s, 57°C for 30 s, and a final step of 72°C for 90 s. COI cycling parameters were 95°C for 10 min, followed by 16 cycles of 94°C for 10 s, 62°C for 30 s (decreasing by 1°C per cycle), 68°C for 60 s, followed by 25 cycles of 94°C for 10 s, 46°C for 30 s, 68°C for 60 s, and a final step of 72°C for 10 min.

PCR triplicates were then pooled and run on a 1.5% agarose gel stained with ethidium bromide to confirm amplification of target genes. The Agencourt AMPure XP bead system (Beckman Coulter) was used to purify PCR products. To confirm removal of excess primers and retention of target amplicons, purified products were run on a second agarose gel. Purified PCR products were quantified using a Qubit dsDNA HS Assay Kit (Invitrogen) and equimolar concentrations of 10 nM per sample were combined into a single gene library pool. Sequencing was performed at the Stanford Functional Genomics Facility on an Illumina MiSeq platform using paired‐end sequencing (Miseq Reagent kit v2) and a 20% PhiX174 spike‐in control to improve the quality of low‐diversity samples (Kircher, Stenzel, & Kelso, 2009).

### 2.4 Bioinformatics and molecular taxonomic identification

Sequence data from this study can be accessed with SRA accession ID SRP134124. A Unix shell script specifically written to analyze Illumina‐generated eDNA metabarcoding data (https://github.com/jimmyodonnell/banzai) was used to process sequence data. The pipeline performs the following main steps: merging of paired reads using PEAR v 0.9.2 (Zhang, Kobert, Flouri, & Stamatakis, 2014), quality filtering with USEARCH (Edgar, 2010), and primer removal with cutadapt v 1.4.2 (Martin, 2011) allowing for no mismatches in the primer sequence. Operational Taxonomic Units (OTUs) were clustered using Swarm, a single linkage clustering algorithm composed of two phases: growth and breaking. During the growth phase Swarm uses a pairwise alignment algorithm to compute differences between aligned pairs of amplicons while the breaking phase refines clustering results by using amplicon abundance information (Mahé, Rognes, Quince, Vargas, & Dunthorn, 2014, 2015). Swarm was chosen to cluster the OTUs because it does not require using a predetermined restrictive percent identity cutoff for OTU assignment, since these cutoffs are highly variable for different molecular markers (Mahé, Rognes, Quince, Vargas, & Dunthorn, 2014, 2015). In order to decrease sequencing errors, sequence reads with homopolymers >7 bases were omitted. Taxonomic annotations for both genes were assigned via the NCBI nt database at 95% similarity to increase the proportion of assigned sequences, with secondary taxonomic assignment using the lowest common ancestor (LCA) algorithm in MEGAN at 70% (Huson, Auch, Qi, & Schuster, 2007). By identifying the lowest common ancestor of the group of taxa a read matched to, the LCA approach increases the number of reads assigned, particularly for less specific matches. Consequently, this means reads with weaker matches to the database will more likely result in assignments at a higher taxonomic level.

To control for contamination, the percent abundance of each OTU that was recovered in the filtration and extraction controls was removed from the samples. First, the average percent abundance of each OTU in the four filtration controls was removed from environmental samples. Subsequently, the percent abundance of each OTU in the extraction blank was removed in a second step. The OTUs from each sample were then filtered and normalized with the R *DESEQ2* package v 1.16.1, which corrects for an uneven sequencing depth across samples, increasing the data stability and focusing the quantitative analysis on the strength of differential abundance (Love, Huber, & Anders, 2014). Statistical analyses were performed in R with the *vegan* package v 2.4–3 (Oksanen et al., 2017). Species richness was obtained by summing a binary presence‐absence matrix of OTUs present in each sample. To evaluate whether species richness was significantly different across months and sites, analysis of variance (ANOVA) followed by Tukey Honest Significant Difference (Tukey HSD) tests were performed. The metaMDS function was used for non‐metric multidimensional scaling (NMDS) analysis to compare community structure among samples based on the binary dataset using Sorenson's distance matrix. A permutational multivariate analysis of variance (PERMANOVA) was calculated using the adonis function to partition the variance between months in the NMDS. Triplicates were then pooled using the merge_samples function within the *phyloseq* package in R (Mcmurdie & Holmes, 2013). Finally, the top 50 genera across all samples were ranked with *phyloseq* and a heatmap was created using the *superheat* R package (Barter & Yu, 2017).

To construct the phylogenetic tree, a consolidated list of all classes identified with either the 18S rRNA and COI gene markers was generated. The tree was created in phyloT using the Newick tree format based on the NCBI taxonomy. The taxa identified by each marker were visualized with the interactive Tree Of Life (Letunic & Bork, 2016).

## 3 RESULTS

### 3.1 Community composition

**Figure 2:**
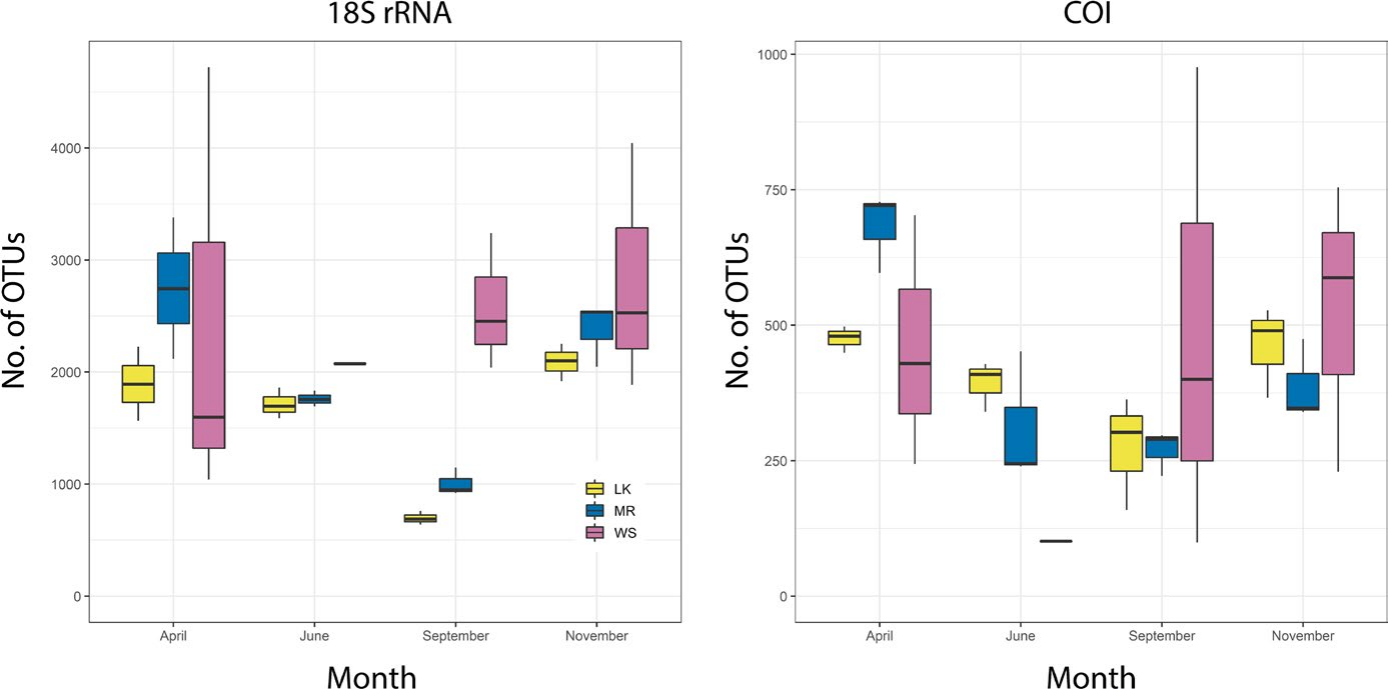
Box‐and‐whiskers plot showing the mean plus/minus the variance of the OTU richness among triplicates for each sample. Months are plotted together, and sites are colored according to the key. LK: Looe Key; MR: Molasses Reef; WS: Western Sambo

The average number of reads recovered for each sample was 113,561 (*SD *= 72,341) for 18S rRNA and 23,085 (*SD *= 9,534) for COI. The average number of reads recovered from the filtration controls was 60,801 (*SD *= 27,529) for 18S rRNA and 13,808 (*SD *= 13,421) for COI, while the average number of reads for the extraction blank was 1,203 for 18S rRNA and four for COI. One of the Western Sambo triplicates in April, September, and November had higher than average reads in both 18S rRNA and COI datasets. 18S rRNA OTU richness was not significantly different between months (ANOVA *p* > 0.05, *df *= 3), but was significantly different between sites (ANOVA *p* < 0.05, *df *= 2; Figure 2). The significance was driven by the difference between richness in Looe Key and Western Sambo in 18S rRNA (Tukey HSD *p* < 0.03). COI OTU richness was not significantly different between months (ANOVA *p* > 0.05, *df *= 3) nor sites (ANOVA *p* > 0.05, *df *= 2). Overall, a higher richness of OTUs was recovered with the 18S rRNA gene, with almost four times more 18S rRNA OTUs compared to COI. The variance of OTU richness among the triplicates in Western Sambo was the highest at all months for both markers.

For the 18S rRNA gene, 16,203 OTUs were recovered, compared to only 3,891 for the COI gene. In comparison, the filtration control recovered 942 OTUs for the 18S rRNA gene and 151 for COI, while the extraction blank recovered 10 and four OTUs for the 18S rRNA and COI genes, respectively. The sequences present in these negative controls were removed from the sample data as described above in the methods. Of the OTUs in the eDNA samples, 52.8% and 21.3% were taxonomically annotated for 18S rRNA and COI, respectively, with 78.9% of 18S rRNA OTUs and 42.8% of COI OTUs annotated to Eukarya (Table 1). The other 21.1% of 18S rRNA OTUs were annotated to Bacteria and Archaea, while the remaining 57.2% of annotated COI OTUs were assigned to “cellular organisms” with only one OTU being assigned to Bacteria. Further analyses focused only on sequences annotated as eukaryotes.

**Table 1:** Breakdown of sequence annotation, pooled across all months and sites for each marker

	Total	Eukarya	Phylum	Class	Order	Family	Genus	Species
18S rRNA								
Sequences	4,097,790	1,888,828	1,810,577	1,702,216	1,335,996	1,335,996	1,164,364	845,798
OTUs	16,203	6,791	6,008	5,442	4,657	4,249	3,887	2,988
Unique	–	–	86	162	376	624	785	918
COI								
Sequences	837,617	425,388	425,327	425,293	425,293	422,475	422,301	418,706
OTUs	3,891	360	358	352	352	336	325	307
Unique	–	–	18	41	75	103	115	125

Note

“Unique” groups refer to the number of taxa that remained after merging OTUs with identical annotation at that taxonomic classification.

COI: cytochrome c oxidase I; OTUs: Operational Taxonomic Units.

**Figure 3:**
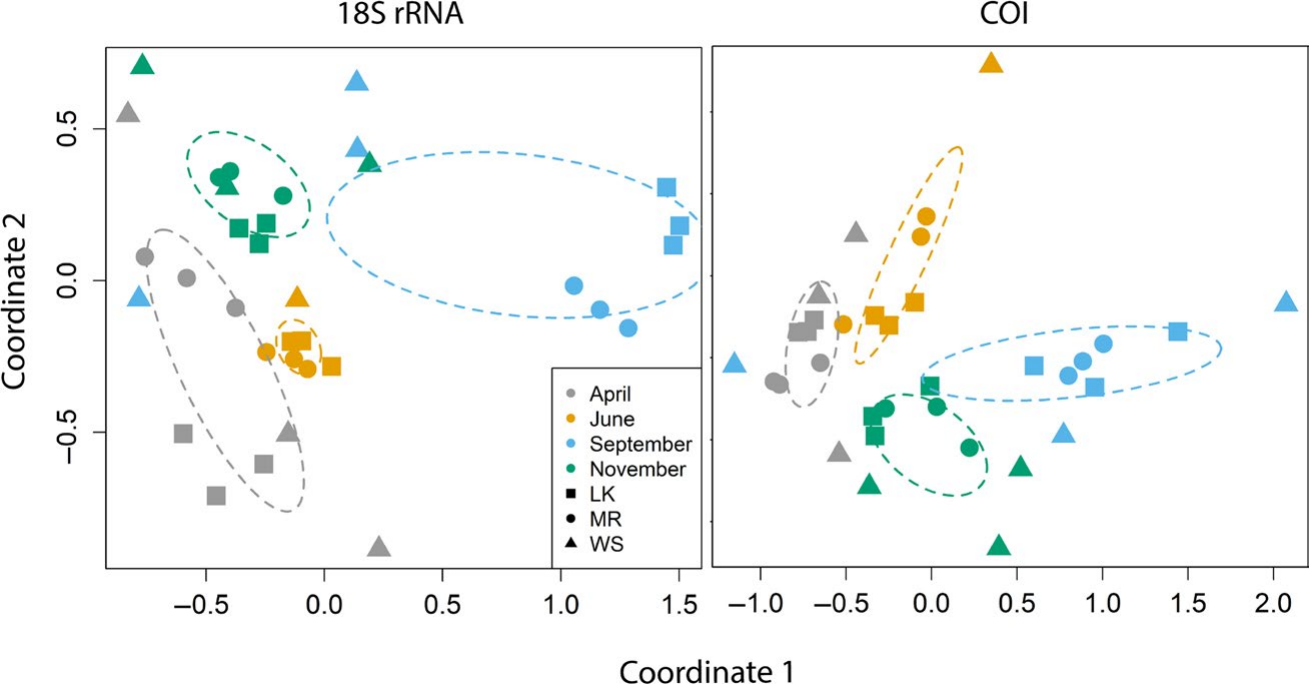
NMDS plots, with a stress of 0.056 for 18S rRNA and 0.11 for COI, showing the similarity of community structures of each sample based on the binary OTU table. Sites are depicted by the different shapes and months are represented by different colors. The ellipses show the 99% standard error of the means based on the centroid calculated for each month. LK: Looe Key; MR: Molasses Reef; WS: Western Sambo

To compare community structure between samples, non‐metric multidimensional scaling (NMDS) was performed based on the presence/absence of each OTU in a sample. The NMDS showed clustering of similar OTU assemblages by seasons (PERMANOVA *p* < 0.05), not by site, for each genetic marker (Figure 3). Most triplicates clustered together on the NMDS plot. Secondary NMDS plots that removed all OTUs found in the negative controls (instead of removing based on proportional abundance as described above) support the patterns observed in the original NMDS, demonstrating that sequences in the controls were negligible with respect to driving community composition (Supporting Information Figure S1).

### 3.2 Major taxonomic groups

**Figure 4:**
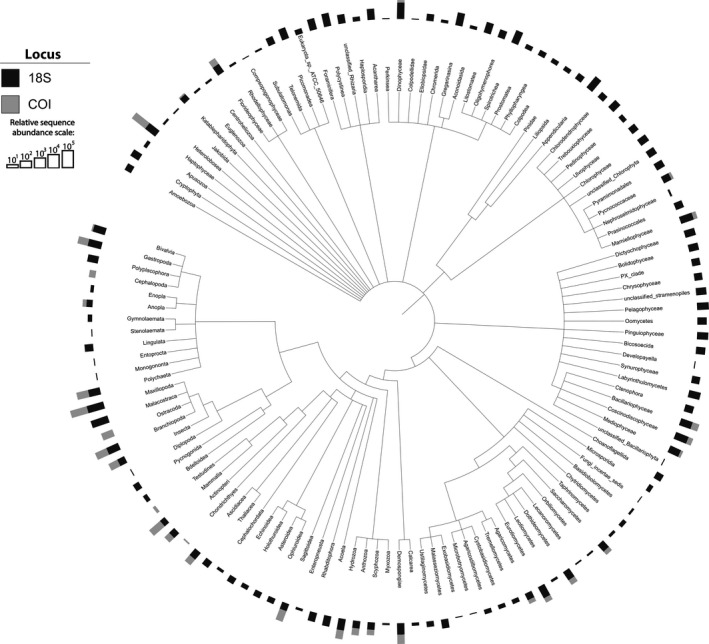
Phylogenetic tree showing the classes recovered by the 18S rRNA and COI markers. Bars above the taxonomy represent the sequence abundance recovered with each marker on a log scale

**Figure 5:**
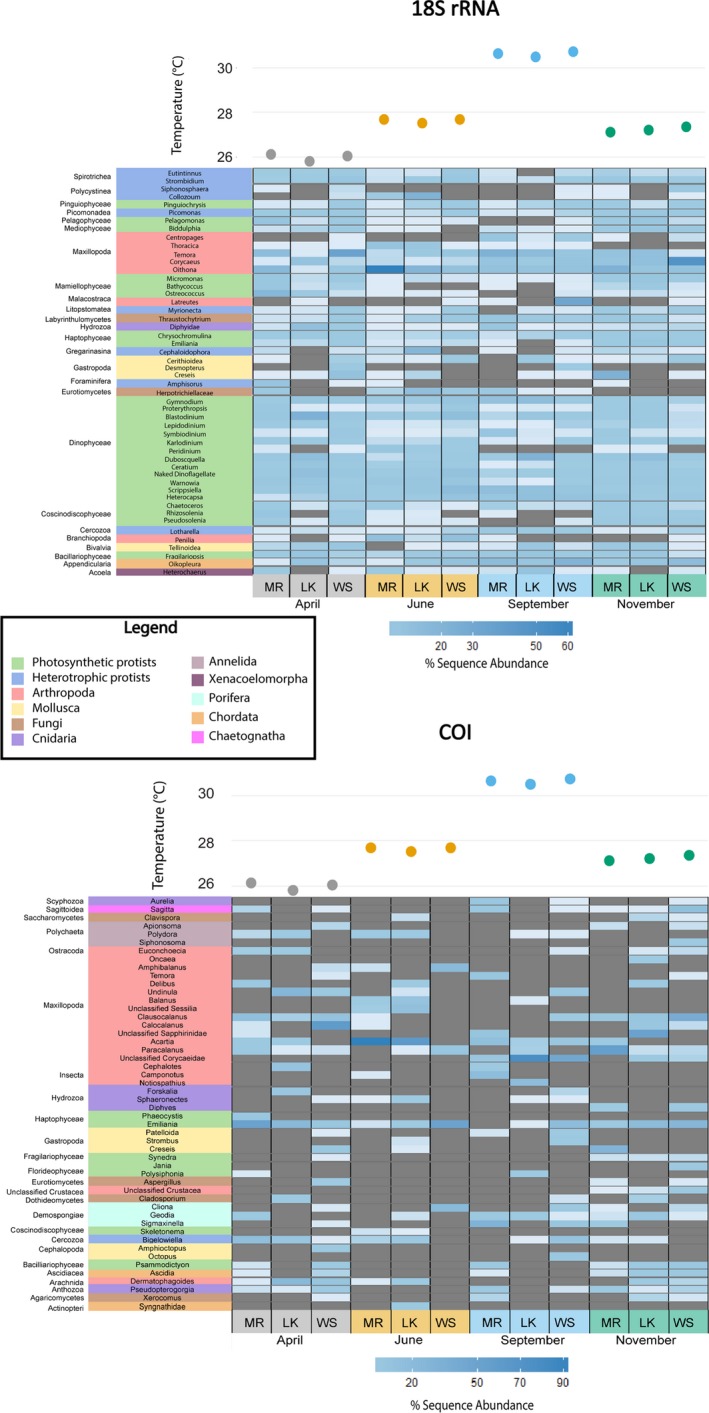
Heatmaps of the percent sequence abundance within each sample of the top 50 genera for each marker. The genera are clustered by class, denoted to the left of the genera. Classes are then clustered into major taxonomic groups by color matching with the legend. Darker blue indicates a higher % sequence abundance, and grey represents the absence of a taxon. Above the heatmaps are the water temperatures (˚C) for each sampling time point and location (LK: Looe Key; MR: Molasses Reef; WS: Western Sambo)

At each classification from phylum to species level, a higher percentage of 18S rRNA OTUs were annotated compared to COI. OTUs with the same taxonomic assignment were merged in the results based on annotations (Table 1, Figures 4 and 5). The number of groups at each classification level, from phyla to species, was consistently higher for 18S rRNA than for COI (Table 1). While both genetic markers recovered the same phyla, at finer taxonomic resolution only 85% of classes, 73% of orders, 51% of families, 29% of genera, and 14% of species identified by COI overlapped with those recovered by 18S rRNA taxonomy.

To compare the taxonomic groups detected by each marker, only OTUs that the pipeline classified at the genus/species level were utilized for the remaining analyses. Comparing the higher‐level taxonomic rankings (i.e., classes) of OTUs that matched these criteria, the 18S rRNA gene identified 130 classes, while only 38 classes were recovered with the COI gene. The phylogenetic tree of classes demonstrates the large overlap in annotated classes from each gene (Figure 4). Five classes were detected only with COI compared to 97 classes detected only with 18S rRNA. Clear differences were seen in the 50 most abundant genera recovered with each genetic marker. Approximately half of the top 50 genera from 18S rRNA were phytoplankton while the top 50 genera from COI consisted primarily of arthropods (Figure 5). The top 50 genera from 18S rRNA were generally ubiquitous across sites and months but the COI genera were more dynamic, with only a few of the top 50 exhibiting widespread distribution (Figure 5). The top 50 genera for each molecular marker were divided into major taxonomic groups, and all genera within these groups were compared between the two markers (details on each group is available in the Supporting Information Appendix S1). Protists and arthropods were the most diverse groups observed regardless of target gene, with over 277 and 85 unique genera recovered, respectively (Table 2). 18S rRNA consistently identified more genera and shared at least one genus with COI in 9 of the 11 biological groups (Table 2). The filtration control and extraction blanks identified 209 genera by 18S rRNA sequencing and 21 genera by COI sequencing. The 18S rRNA filtration control was dominated by genera within Polycystinea, Lingulata, Hydrozoa, and Insecta. The COI filtration control was primarily dominated by *Homo sapiens* and the copepod genus *Clausocalanus* (Supporting Information Figure S2, Table S1). Reads present in the controls could represent potential contamination and thus were removed from the samples as described above in the methods section.

**Table 2:** A breakdown of the number of genera recovered from the major taxonomic groups for 18S rRNA, cytochrome c oxidase I (COI) and both markers

Major group	18S rRNA	COI	Shared
Photosynthetic protists	201	19	14
Heterotrophic protists	70	1	0
Arthropoda	64	31	10
Mollusca	45	9	4
Fungi	56	6	3
Cnidaria	38	10	2
Annelida	44	6	3
Xenacoelomorpha	1	0	0
Porifera	42	12	5
Chordata	22	6	1
Chaetognatha	3	1	1

Note

Only major groups represented in the the top 50 genera for either marker are included in the table.

## 4 DISCUSSION

### 4.1 Community composition

Consistent with previous studies, it is clear that metabarcoding of multiple molecular markers recovers different taxa and thus a wider variety of taxa, making it more informative for assessing biodiversity than using a single marker (Kelly et al., 2017; Stat et al., 2017). Although sequencing of the 18S rRNA gene detected more OTUs than the COI gene and included all the phyla found with COI, it failed to recover genera and families that were abundant in COI. Thus, only using one marker gene would have yielded a skewed view of biodiversity. In fact, using only two markers is still limiting, and we would likely get a more comprehensive view of the ecosystem by adding markers such as 12S rRNA to selectively recover vertebrates (Andruszkiewicz et al., 2017; Port et al., 2016; Riaz et al., 2011). Although 18S rRNA and COI can recover chordates, they make up a small portion of the sequences. Both the 18S rRNA and COI datasets were overwhelmed by phytoplankton and arthropod sequences due to their higher abundance in eDNA.

Due to limitations of taxonomic representation in the databases, some species were annotated to the most closely related representatives, resulting in misidentification of species that are not known in the Gulf of Mexico. For example, the only *Polydora* species annotated, *Polydora ciliate*, is not known to reside in the Gulf of Mexico. Recovery of *Heterochaerus australis* is perhaps another example of a database limitation, particularly since the closely related species *Heterochaerus sargassi* occurs in the west Atlantic and thus is more likely to be in the FKNMS. In addition, the annotations for the Polyplacophora (chiton) species identified (*Katharina tunicata, Tonicella lineata, Mopalia muscosa, Stenoplax alata,* and *Plaxiphora albida*) are doubtful as they all occur in the Pacific Ocean, native to Australia, Russia, and North America (Horton et al., 2018). In all these cases, there are related species found in Florida and the Caribbean, but the lack of representation of these species in public sequence databases likely explains the annotation to closely related species found elsewhere. Alternatively, due to the limitations of visual surveys (including microscopy) it is also possible that some taxa have been overlooked visually but are now being detected through eDNA metabarcoding (Djurhuus et al., 2018; Kelly et al., 2017).

While richness was similar among months, the NMDS and patterns in arthropod diversity showed greater influence of temporal differences than spatial differences on community structure. Richness was significantly different between Looe Key and Western Sambo in the 18S rRNA data, which is interesting as those are the two locations closest geographically, emphasizing the importance of conducting biodiversity surveys at differing spatial scales to recover broadly distributed as well as localized taxa. Sequences from both genetic markers were dominated by phytoplankton and zooplankton, and thus it is credible that these communities vary temporally with changing temperature, light availability, and nutrient concentrations (Tilman, Kilham, & Kilham, 1982). It is unlikely that the presence of sessile organisms, such as sponges and corals, are changing drastically throughout the seasons, explaining why less of a seasonal trend is evident when focusing on these trophic groups/classes individually. On occasion; however, differences were seen in the recovery of sessile organism sequences from a single location in different months. The sponge *Ircinia felix*, for example, was identified at all locations but not detected in all months (Supporting Information Appendix S1). Potential explanations include spawning or physiological changes associated with season and/or organism health since eDNA shedding rates are dependent on life stage and influenced by stress (Maruyama, Nakamura, Yamanaka, Kondoh, & Minamoto, 2014; Pilliod, Goldberg, Arkle, & Waits, 2013).

### 4.2 Limitations and advantages of eDNA

Given the increased use of eDNA metabarcoding for monitoring biodiversity in the marine realm, it is important to consider the advantages and limitations of this technique. A major limitation of eDNA metabarcoding is that the data can only indicate the presence, but not absence of a species, as recovery is dependent on primer bias, sequencing depth, and eDNA shedding rates. In addition, relative sequence abundance does not necessarily reflect the number organisms or their biomass in a given sample. For example, our results show that smaller pelagic organisms dominate the sequences, which could potentially result from direct capture of these organisms on the filter. For example, 18S rRNA sequences from the protist *Collozoum amoeboides* were abundant in April at Looe Key, with close to tenfold as many sequences from this species compared to any other sample or any other protist group. A potential explanation might be the colonial nature of the genus *Collozoum*, which may have resulted in capturing multiple cells in one sample. Resting, vegetative, parasitic, symbiotic, and/or sexual stages can also influence copy number of the target marker, in addition to inherent differences across phyla and organisms of different sizes. Using biomass conversions could aid with quantifying eDNA, and there have been studies that have had success doing so (Goldberg, Pilliod, Arkle, & Waits, 2011); however, performing these calculations becomes more difficult when looking at broader groups across trophic levels and using multiple marker genes (Djurhuus et al., 2018). It is also essential that environmental variables such as temperature and ultraviolet light, which affect the stability of eDNA, are taken into consideration when using biomass conversions.

Another limitation of eDNA is the inability to distinguish between living and dead organisms, or to identify the life stage of the organism that was recovered (Rees, Maddison, Middleditch, Patmore, & Gough, 2014). For example, an organism may release a large amount of eDNA into the water column as the result of predation, resulting in detection of an organism that is no longer present. Finally, the presence of ocean circulation and currents can confound the origin of the DNA sample, and although often the genetic material degrades before being transported too far from the source (Kelly, Port, Yamahara, & Crowder, 2014; O'Donnell et al., 2017; Thomsen et al., 2012) many organisms have life cycle stages with the capacity for dispersal across varied spatiotemporal scales (Green et al., 2015; Marcus & Boero, 1998; Palumbi, 2004; Shanks, Grantham, & Carr, 2003). These facts emphasize that although eDNA has the potential of providing an enormous amount of information about an ecosystem, traditional surveys have distinct value and should continue in conjunction with eDNA surveys for more robust and accurate assessments of biodiversity (Djurhuus et al., 2018; Kelly et al., 2017). In fact, visual surveys can detect species missed by eDNA, which is something to keep in mind when using eDNA as a biodiversity monitoring tool (Djurhuus et al., 2018; Kelly et al., 2017). This discrepancy can be partly due to primer bias and the limitations of the number of distinct species a primer can recover (Parada, Needham, & Fuhrman, 2016; Stat et al., 2017). However, another reason for this is likely because of an incomplete database of marine organism sequences for the target genes (Djurhuus et al., 2018; Kelly et al., 2017).

Although target genes are specifically selected because they are commonly used during DNA barcoding, there are still large gaps in the database for certain organisms (Kelly et al., 2017), as evident by the large number of unassigned OTUs for both molecular markers (Table 1). To compare results between genetic markers, both datasets in this study were compared to the NCBI nt database, even though SILVA is the preferred database for 18S rRNA gene analyses and BOLD is the preferred database for COI gene analyses (Bik et al., 2012; Pruesse et al., 2007; Ratnasingham & Hebert, 2007). Comparison of the 18S rRNA sequences to SILVA only resulted in 8% more OTUs annotated than was achieved with the NCBI nt database; nevertheless, curated sequence databases are of great utility especially given the potential for taxonomic ambiguity and errors in public databases. This increase in annotation by SILVA is due to the manual curation of the database, which leads to assignments of sequences that had matches in NCBI but could not be given taxonomic assignments due to multiple hits that had identical scores and poor classification, resulting in different annotations and to different levels. These hits often include environmental sequences that are annotated poorly, giving little insight into taxonomic classification. In SILVA, these duplicates have been removed from the database and only reference sequences with clear taxonomic classifications have been retained, allowing clear assignment. In contrast, the percent of sequences with “no hits” (i.e., those that did not share >95% identity to any reference sequence in the database) was higher during comparison against SILVA than NCBI, due to the removal of poorly annotated environmental sequences. As studies expand the use of metabarcoding to analyze community composition, it is essential to continue to prioritize the addition of reference sequences that will permit an increased number of annotated sequences and a more comprehensive view of biodiversity in future monitoring surveys. This will likely become less of a hindrance over time as more voucher specimens are sequenced, creating more robust databases. Since sequences can be reanalyzed against improved databases in the future, eDNA metabarcoding is highly advantageous for time‐series studies focused on observing long‐term changes of community structure in an ecosystem because historical data can be analyzed in conjunction with new data and updated reference databases.

As demonstrated in this study, eDNA allows for simultaneous monitoring of multiple trophic levels, as opposed to visual surveys and other traditional sampling techniques that often focus on one trophic level or even more specifically one taxon. This enables the evaluation of ecosystem dynamics as a whole and permits the monitoring of co‐occurrence patterns between and among taxa. In comparison to traditional techniques, which mostly rely on visual identification, eDNA allows for a more rapid sampling effort (i.e, dive survey vs. filtration) and analysis (i.e., taxonomic identification vs. sequencing) with respect to the large amount of species recovered (Kelly et al., 2017). Another advantage is that collecting samples for eDNA analyses is non‐invasive and does not harm or disturb the organisms of interest (Rees et al., 2014). Since filtration of one liter of water is straightforward, ancillary eDNA samples can easily be collected in conjunction with traditional surveys to compare techniques and provide essential baseline information for future studies aimed at detecting ecosystem change.

Metabarcoding of eDNA is a useful tool for observing biodiversity across multiple trophic levels and can provide insight into a community from a single, small‐volume water sample. However, eDNA metabarcoding is not without its limitations; it is neither quantitative nor can it identify life stages of organisms. The primers themselves also have biases and thus using more gene targets is preferable when trying to maximize taxa recovery. The large number of unannotated sequences resulting from gaps in the database also hinders achieving a complete view of ecosystem community structure, though the utility of eDNA metabarcoding analysis will improve as more voucher sequences become available. Despite these limitations, this study recovered over 1,000 species across multiple trophic levels, thus emphasizing the potential of eDNA metabarcoding to detect a wide range of biodiversity from a single liter of sea water. These results highlight the potential for eDNA metabarcoding to be used as a monitoring tool in the FKNMS, and inform future studies needed to establish effective long‐term biodiversity monitoring systems.

## CONFLICT OF INTEREST

None declared.

## AUTHOR CONTRIBUTIONS

NAS and AD collected and processed the samples. NAS analyzed the data and took lead on writing the manuscript with support from AD and MB. All authors contributed to analyses and interpretation of results in their respective expertise. CJC provided Figure 4 and molluscan analyses. MH provided chordate analyses. LV provided Figure 1 and aided with sample collection. EO and KH provided phytoplankton analyses. CK provided arthropod analyses. MB supervised the project.

## Data Availability

Raw sequence data from this study can be accessed at NCBI with SRA accession ID SRP134124.
