# Supplementary figures and images for: Assessing eukaryotic biodiversity in the Florida Keys National Marine Sanctuary through environmental DNA metabarcoding
Source: Ecol Evol. 2019 Jan 15;9(3):1029–40. doi: 10.1002/ece3.4742 (PMC6374654; doi:10.1002/ece3.4742)

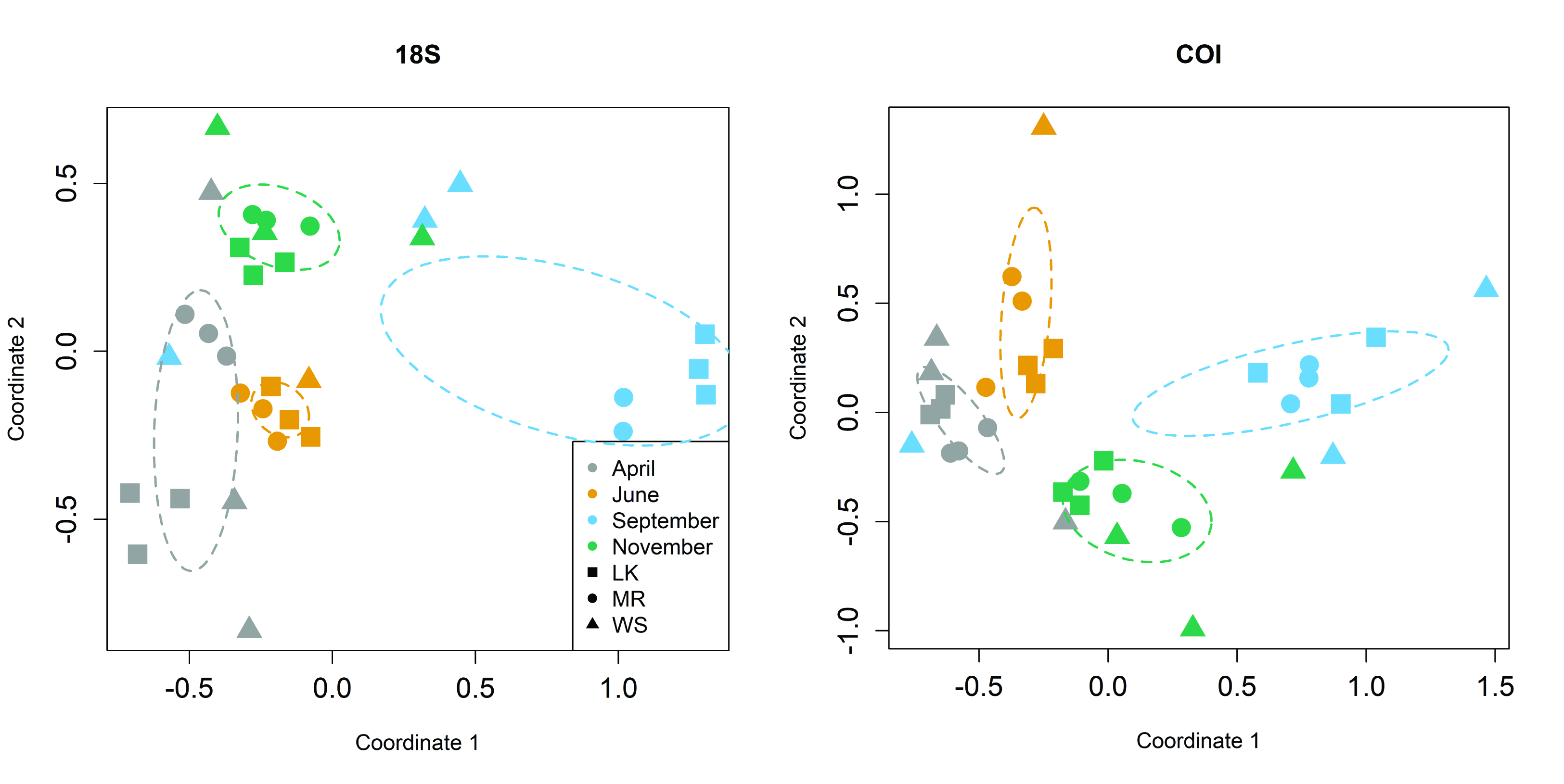

Supplement: Supplementary file 1 [file ECE3-9-1029-s001.tif]

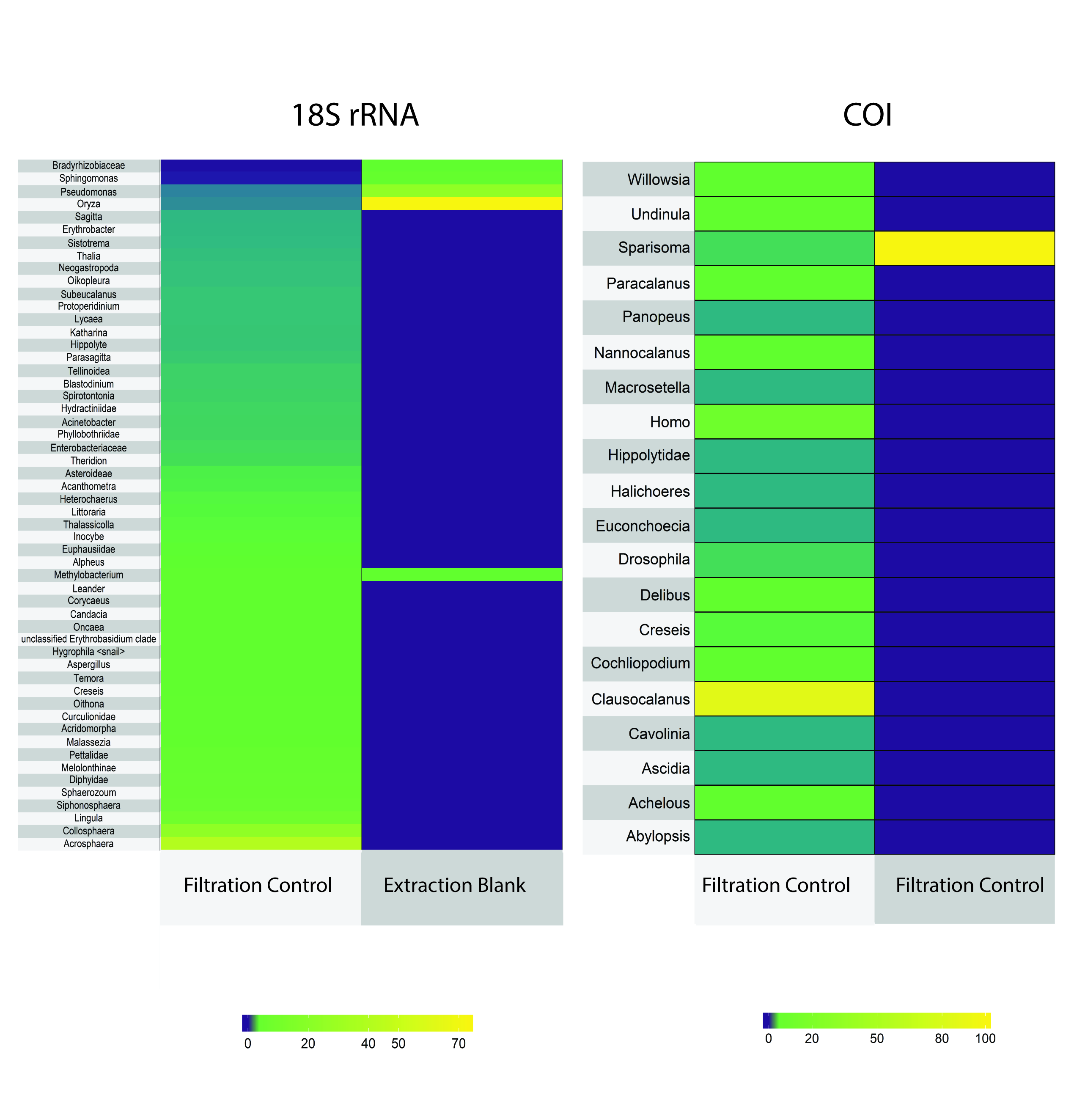

Supplement: Supplementary file 2 [file ECE3-9-1029-s002.tif]
