# Supplementary material for: Assessing eukaryotic biodiversity in the Florida Keys National Marine Sanctuary through environmental DNA metabarcoding
Source: Ecol Evol. 2019 Jan 15;9(3):1029–40. doi: 10.1002/ece3.4742 (PMC6374654; doi:10.1002/ece3.4742)
Supplement: Supplementary file 3 [file ECE3-9-1029-s003.docx]

Supporting Information

Appendix S1

Photosynthetic protists

Seven phytoplankton phyla were represented within the 18S rRNA dataset. Of those, five were also recovered by COI sequencing. The dominant phyla varied depending on which marker was examined. For example, COI sequences were dominated by haptophytes whereas 18S rRNA sequences were dominated by dinoflagellates. In addition, 18S rRNA sequencing revealed 201 phytoplankton genera (31.4% of the total 18S rRNA taxonomic assignments) compared to only 19 genera identified with COI (9.7% of COI taxonomic assignments). Five genera were recovered only by COI sequencing, while the other 14 COI genera were also identified by 18S rRNA, including members of the red algae (Rhodophyta), green algae (Chlorophyta), diatoms (Bacillariophyta), haptophytes (Haptophyta), and dinoflagellates (Myzozoa).

The COI dataset was dominated by the cosmopolitan coccolithophore, *Emiliania huxleyi,* which was four times more abundant than all other taxa combined. This alga was the 17^th^ most abundant taxon in the 18S rRNA dataset and was either not detected or present at very low levels in the September Looe Key and Molasses Reef samples for both markers. The next most abundant taxon observed via COI was the globally distributed benthic diatom, *Psammodictyon panduriforme,* previously described in association with coral reefs in the Western Indian Ocean (Al-Handal *et al.* 2016) as well as other substrates. This alga was not detected by 18S rRNA, however, only partial sequences covering the 18S rRNA amplicon are currently available in public databases. With nine OTUs and each of the four Bacillariophyta orders observed, diatoms were the richest group detected with COI (compared to dinoflagellates for 18S rRNA), followed by the multicellular Rhodophyta (red algae). The red algae spanned four (COI) to seven (18S rRNA) orders, with both markers detecting members of the Corallinales. Corallinales form calcareous deposits and are commonly found in association with corals, where they can serve as a food source to reef-dwelling biota. Roughly half of 28 rhodophyte genera revealed with 18S rRNA were previously identified in the FL Keys (FKNMS Comprehensive Management Plan), compared to all six of the COI genera, yet the COI marker recovered an additional three genera, as well as two OTUs for *Polysiphonia* (compared to only one for 18S rRNA). For both markers, the few rhodophyte sequences that could be identified to species level did not correspond with previously observed species in the FKNMS, with the exception of two 18S rRNA OTUs, *Jania rubia* and *Crouania attenuata*.

In general, however, the 18S rRNA phytoplankton dataset revealed a comparatively richer assemblage than the COI marker, with two additional phyla recognized: Ocrophyta (which includes the Dictyochophyceae, Chrysophyceae, Pelagophyceae and Pinguiophyceae classes) and Euglenozoa. The top 50 phytoplankton genera identified with 18S rRNA included members of the Myzozoa, Haptophyta, Bacillariophyta, Chlorophyta, and Ocrophyta, with >20 of the top 50 18S rRNA phytoplankton genera observed in all samples. Sixty-one genera spanning twelve orders within the most diverse class, Dinophyceae, were observed with 18S rRNA, with 28 genera represented in the top 50. Nine of the ten most abundant genera were dinoflagellates; the globally important picoplankton, the chlorophyte *Ostreococcus,* ranked 6^th^. The cosmopolitan dinoflagellate *Scrippsiella* was most abundant genus identified by 18S rRNA, representing 13.3% of phytoplankton reads and 2-27% of total reads from each sample. Interestingly, the representative sequence for this OTU was 100% identical to a very closely related freshwater dinoflagellate genus that contains only a single species, *Theleodinium calcisporum*, and only had a single mismatch with sequences from different *Scrippsiella* spp. Although *Scrippsiella* is commonly observed in Florida’s marine waters (FWC Harmful Algal Bloom Historical Monitoring Database), *Theleodinium* has only been observed in freshwater systems in Portugal based on the original and only description of this genus (Craveiro *et al.* 2013); both genera share the ability to form calcareous cysts. Although the sequence obtained might be due to novel marine species of *Theleodinium, Scrippsiella,* or other closely related genera, the bioinformatics pipeline assigned this dinoflagellate taxon to the genus *Scrippsiella*. Corresponding sequences represented >15% of total sequences in June samples from Looe Key and Western Sambo, and September samples from Western Sambo and Molasses Reef, which was notable given the overall high richness of samples. The second and third most abundant genera, the thecate dinoflagellate *Heterocapsa* and the athecate dinoflagellate *Warnowia*, respectively, also form cysts. These top three algae represented 10 to 40% of sequences across samples, with the highest proportion observed in September at Western Sambo.

The 19^th^ most abundant genus was *Symbiodinium*, a dinoflagellate that has free-living stages and lives in symbioses with a wide range of hosts including invertebrates (most notably corals) and protists (e.g., Stat *et al.* 2006, Kemp *et al.* 2015). This dinoflagellate was observed in all samples but was more prevalent at Molasses Reef during September/November, and at Western Sambo in April. Several dinoflagellate genera that contain harmful species – able to produce toxins that harm humans and/or wildlife and therefore of interest to Sanctuary managers – were also identified, including *Karlodinium, Alexandrium, Takayama, Prorocentrum, Gonyaulax, Cochlodinium, Dinophysis, Akashiwo, Azadinium,* and *Ostreopsis;* the first five were among the top 50 18S rRNA genera. Other notable taxa among the top 50 18S rRNA genera include 15 pico- and nanoplankton (which have smaller genome sizes than dinoflagellates) from the Ocrophyta, Chlorophyta, and Haptophyta phyla, and 10 diatom genera, including the toxigenic genus *Pseudo-nitzschia* (also detected by COI).

Nearly all of the top 50 genera are known to occur in Florida based on prior microscopy analysis (FWC Harmful Algal Bloom Historical Monitoring Database), with the exception of some of the endoparasitic dinoflagellates which were in general, well-represented throughout the 18S rRNA data. These genera contain species that can parasitize marine ciliates, other dinoflagellates, and/or copepods, and also have free-living stages (e.g., *Duboscquella, Blastodinium, Amoebophrya*). There is evidence that suggests that parasite-host relationships can be species specific (Skovgaard *et al.* 2012), and both *Blastodinium* and *Amoebophyra* were among the most OTU-rich genera. Three of the six *Blastodinium* species recovered co-occurred with sequences from their copepod hosts (Skovgaard *et al.* 2012). The copepod *Clausocalanus furcatus* is a host to all three of these *Blastodinium* species (*B. contortum, B. spinulosum,* and *B. inornatum*) and was recovered in eight of the 12 sampling events; each of these times, at least one of these parasitic dinoflagellate species was also present.

Heterotrophic protists

Since a vast diversity of protists was detected, only the four heterotrophic protistan phyla (Polycystinea, Ciliophora, Cercozoa, and Apicomplexa) that contained members in the top 50 genera for either genetic marker will be discussed in this section. While all four of these phyla were recovered with 18S rRNA, only genera from Polycystinea and Ciliophora were present in the 18S rRNA top 50. Of these four phyla, 18S rRNA detected 70 unique genera and COI only detected one species, *Bigelowiella natans*, which is within the Cercozoa. *Bigelowiella natans*, which was only recovered with COI, was most abundant in April and June for both Looe Key and Molasses Reef but was most abundant at Western Sambo in November. *Collozoum*, a genus within Polycystinea, dominated the protistan 18S rRNA sequences at Looe Key in June; however, this genus was rare in all other samples. In contrast, *Strombidium*, a genus within Ciliophora, had the second highest number of sequences within the protists and was detected more consistently between samples, peaking in April at Western Sambo.

Arthropoda

Sixty-four unique genera of Arthropoda were recovered with 18S rRNA and 31 unique genera with COI. OTUs assigned to *Temora* spp., *Oithona hebes*, *Oithona sp. 2 New Caledonia-RJH-2004*, and *Corycaeus speciosus* were recoveredin all samples by18S rRNA, and OTUs assigned to *Euterpina acutifrons*, *Oncaea* sp., and *Thoracica* sp. were recoveredin at least 75% of the 18S rRNA samples. In the COI data, the most commonly assignedspecies were *Paracalanus quasimodo*, *Acartia tonsa*, and *Clausocalanus furcatus*. *Paracalanus*, *Acartia*, *Eutrepina*, *Temora*, and *Oithona* are all known to be common copepod genera in the Florida Keys and adjacent water bodies (Heidelberg *et al.* 2010; Kelble *et al.* 2010). *Corycaeus speciosus* and *Oncaea* are common in tropical, neritic waters of the Atlantic, including the Florida Keys (López-Salgado & Suárez-Morales 1998; Fernández-Álamo *et al.* 2000; Heidelberg *et al.* 2010). The order Thoracica, found in the 18S rRNA data, is known to be a common barnacle taxon in the Gulf of Mexico (Gittings 1985).

None of the arthropod species in either the 18S rRNA or COI data were identified exclusively at one location. There were some consistencies in the arthropods present within monthly samples for some species. In the 18S rRNA data, *Oncaea* sp. was not detected at any location in June, but present in all other samples. *Thoracica* was not recovered with 18S rRNA at any stations in November but was present in all other samples. Sequences annotated as *Acartia tonsa* were present in the 18S rRNA data at all locations in June, but only present in one other sample in the study. In the COI data, *Clausocalanus furcatus* was present at all stations in April and November, but only present at one station each in June and September. *Corycaeidae sp. Co432.1.3* was present in the COI data at all stations in November and all but one in September but was not present at any of the stations in April and June. These patterns suggest that temporal changes in arthropod diversity are greater than spatial variability.

Mollusca

Under the Phylum Mollusca, all four extant epipelagic classes (Bivalva, Cephalopoda, Gastropoda, and Polyplacophora) were identified with the combination of both markers. Cephalopoda was not detected in the 18S rRNA sequences, while COI identified all but Polyplacophora. In total, 18S rRNA identified 45 genera and COI identified 9 genera. Four genera, *Creseis*, *Strombus*, *Patelloida*, and *Limaria* were recovered with both markers. The only molluscan species identified with both markers was *Patelloida pustulata*, the true limpet.

Fungi

Fifty-six genera of fungi were recovered with 18S rRNA, while COI only detected 6 genera. Three genera (*Clavispora*, *Penicillium*, and *Aspergillus*) overlapped between the two markers. The dominant species from COI was *Cladosporium tenuissimum*, which is the most common spore found in air and often causes allergies. This species was not detected with 18S rRNA. The five most abundant taxa, representing 87% of all reads assigned to fungi with 18S rRNA, were, in order, Herpotrichiellaceae, *Malassezia globosa*, unclassified Dothideales, unclassified Pleosporaceae, *Cyberlindnera jadinii*. All five taxa were present at all locations with the highest abundance during June and September.

Cnidaria

Four classes of cnidarians were identified with 18S rRNA (Hydrozoans, Anthozoans, Myxozoans, and Scyphozoans), while all but Myxozoans were recovered with COI. Five orders of hydrozoans (Siphonophorae, Anthoathecata, Trachymedusae, Leptothecata, and Narcomedusae) were recovered with 18S rRNA, two of which (Siphonophorae and Leptothecata) were also identified with COI. *Diphyes*, a type of siphonophore, was the most abundant cnidarian genus across all sites and months combined for both the 18S rRNA and COI. Overall, 38 cnidarian genera were recovered with 18S rRNA and 10 cnidarian genera were recovered with COI. *Zanclea*, a genus within Hydrozoans, was the second most abundant cnidarian detected with 18S rRNA and the sequence was 100% identical to *Zanclea* sp. ZX JRH-2014. Although the exact relationship remains unclear, *Zanclea* are known to be symbiotic with reef-building corals in the order Scleractinia, which were also recovered with 18S rRNA (Pantos & Bythell 2010). This finding is interesting since *Zanclea* have primarily been known to reside in the Indo-Pacific region but recently identified in the Caribbean Sea and the Atlantic Ocean (Montano *et al.* 2017). *Zanclea* was only abundant in Looe Key during June, as opposed to *Diphyes* which was abundant in multiple months at various locations. Only one order of Scyphozoans, Semaeostomeae, was obtained with COI while 18S rRNA also recovered the order Coronatae. Within Anthozoa both markers recovered the order Alcyonacea, which contains soft corals. Although samples were collected at the surface, benthic organisms such as sea anenomes, stony corals, and soft corals were recovered through eDNA. However, the sequences recovered were dominated by pelagic species, as demonstrated by the large number of siphonophores in both 18S rRNA and COI sequences.

Annelida

All Annelida sequences from both markers belong to the Class Polychaeta; 44 genera were recovered with 18S rRNA and 6 genera with COI. Three genera of burrowing polychaetes known to inhabit the Gulf of Mexico (*Apionsoma*, *Polydora*, and *Eunice*) were recovered with both markers (Dauer 1973, Dean 2002). The FKNMS sampling sites were fairly shallow (~ 20 m), so it is possible that eDNA from benthic organisms was present in surface waters; however, the eDNA may also originate from spawning of their gametes.

Xenacoelomorpha

*Heterochaerus australis*, from the phyla Xenacoelomorpha, was found in 10 of the 12 sampling events. *Heterochaerus australis* was only recovered with 18S rRNA and has been previously observed in the Indian Ocean (Horton *et al.* 2018).

Porifera

Two classes of Porifera (Demospongiae and Calcarea) were recovered with 18S rRNA, while only Demospongiae were recovered with COI. 18S rRNA detected 42 sponge genera, while COI only detected 12 genera. Most 18S rRNA sponge sequences were from Molasses Reef and Western Sambo. Despite having more sequences, none of the sponges belonged to the top 50 genera detected from 18S rRNA, while three genera of sponges (*Cliona*, *Geodia*, and *Sigmaxinella*) were in the top 50 genera recovered with COI (Figure 5). Though dominating COI, *Cliona*, *Geodia*, and *Sigmaxinella* were not detected in 18S rRNA sequences. The most abundant 18S rRNA sponge sequence was from the species *Ircinia felix*, which was found at all locations. This species is known to inhabit Florida reefs and often grows near coral; thus, it is not surprising that it was found across all three sites as they are near coral reefs (Duchassaing de Fonbressin & Michelotti 1864). Despite their widespread presence, *Ircinia felix* sequences were not detected from Looe Key in April or September or from Molasses Reef in September. Since *Ircinia felix* is a sessile sponge species, its presence at a given location does not change over time, suggesting there is another explanation for the variability in detection over time. It is possible that the eDNA detected from sponges at the surface originated from spawning events; however, since both instances where sponges were not detected occurred in September where the temperature is the highest, it is also possible that higher DNA degradation rates contributed to the lack of detection of *Ircina felix* sequences (Strickler *et al.* 2015).

Chordata

A total of 28 chordate genera were detected across sampling sites and months. Of the 22 genera detected with 18S rRNA, 18 were in the subphylum Tunicata (14 of class Ascidiacea and four of class Thaliacea). The *Pagrus* and *Oikopleura* genera were the most abundant Chordata detected with 18S rRNA and these genera were detected in all of the samples. 18S rRNA sequences also included *Branchiostoma floridae* (Florida lancelet) a benthic species often found in shallow waters of the Gulf of Mexico (Horton *et al.* 2018). Additionally, the COI detected six chordate genera, which included benthic, demersal, and pelagic species. For example, the COI detected *Lonchopisthus micrognathus* (swordtail jawfish) often found on muddy bottoms, as well as the *Chelonia mydas* (green sea turtle) and *Carcharhinus perezii* (Caribbean reef shark), which migrate between benthic feeding grounds in (e.g., seagrass beds and coral reefs) to the pelagic continental shelf. Overall, very few chordate species were detected with these two genetic markers and the results do not adequately reflect the taxonomic composition of the vertebrate community along the FKNMS reef tract.

Chaetognatha

Chaetognatha were identified with both genetic markers, with the 18S rRNA data recovering three genera (*Sagitta*, *Eukrohnia*, and *Parasagitta*) and the COI recovering two species belonging to the genus *Sagitta*. All genera detected in this study are commonly found in the Gulf of Mexico (Felder *et al.* 2009). The genus *Eukrohnia*, most commonly found in the deep sea and known for its bioluminescence, was only found at Western Sambo (Thuesen *et al.* 2010). The genus *Sagitta*, which is the most abundant pelagic group in the Chaetognatha phylum, was present in all locations and all months except for June for both loci. The two taxa in the genus *Sagitta* from COI were annotated to species *Sagitta helenae* and *Sagitta enflata* (recently renamed *Flaccisagitta enflata*), which was the most abundant chaetognath taxa from COI and dominated the Chaetognatha in November at all locations.

References

Al-Handal AY, Pennesi C, Abdullah DS (2016) *Mastogloia abnormis* sp. nov. and *Mastogloia descrepata* sp. nov.(Bacillariophyceae, *Mastogloia* section Sulcatae) from Sawa Lake, Southern Iraq. *Diatom Research*, **31**, 113–121.

Craveiro SC, Pandeirada MS, Daugbjerg N, Moestrup Ø, Calado AJ (2013) Ultrastructure and phylogeny of *Theleodinium calcisporum* gen. et sp. nov., a freshwater dinoflagellate that produces calcareous cysts. *Phycologia*, **52**, 488–507.

Dauer DM (1973) Polychaete fauna associated with Gulf of Mexico sponges. *Florida Scientist*, **36**, 192–196.

Dean HK (2002) Marine biodiversity of Costa Rica: the phyla Sipuncula and Echiura. *Revista de Biología Tropical*, **49**, 85–90.

Duchassaing de Fonbressin P, Michelotti G (1864) *Spongiaires de la mer Caraïbe*. Les héritiers Loosjes.

Felder DL, Camp DK, Tunnell Jr JW (2009) An introduction to Gulf of Mexico biodiversity assessment. In: *Gulf of Mexico Origin, Waters, and Biota*, **1**, 1–13.

Fernández-Álamo MA, Sanvicente-Añorve L, Alameda-De-La-Mora G (2000) Copepod assemblages in the Gulf of Tehuantepec, Mexico. *Crustaceana*, **73**, 1139–1153.

Gittings SR (1985) Notes on barnacles (Cirripedia: Thoracica) from the Gulf of Mexico. *Gulf and Caribbean Research*, **8**, 35–41.

Heidelberg KB, O’neil KL, Bythell JC, Sebens KP (2010) Vertical distribution and diel patterns of zooplankton abundance and biomass at Conch Reef, Florida Keys (USA). *Journal of Plankton Research*, **32**, 75–91.

Horton T, Kroh A, Ahyong S, Bailly N, Boury-Esnault N, Brandão SN, Costello MJ, Gofas S, Hernandez F, Mees J, Paulay G, Poore GCB, Rosenberg G, Decock W, Dekeyzer S, Lanssens T, Vandepitte L, Vanhoorne B, Verfaille K, Adlard R, Adriaens P, Agatha S, Ahn KJ, Akkari N, Alvarez B, Anderson G, Angel M, Arango C, Artois T, Atkinson S, Bank R, Barber A, Barbosa JP, Bartsch I, Bellan-Santini D, Bernot J, Berta A, Bieler R, Blanco S, Blasco-Costa I, Blazewicz M, Bock P, Böttger-Schnack R, Bouchet P, Boxshall G, Boyko CB, Bray R, Breure B, Bruce NL, Cairns S, Campinas Bezerra TN, Cárdenas P, Carstens E, Chan BK, Chan TY, Cheng L, Churchill M, Coleman CO, Collins AG, Corbari L, Cordeiro R, Cornils A, Coste M, Crandall KA, Cribb T, Cutmore S, Dahdouh-Guebas F, Daly M, Daneliya M, Dauvin JC, Davie P, Broyer C De, Grave S De, Mazancourt V de, Voogd N de, Decker P, Decraemer W, Defaye D, d’Hondt JL, Dijkstra H, Dohrmann M, Dolan J, Domning D, Downey R, Drapun I, Ector L, Eisendle-Flöckner U, Eitel M, Encarnação SC d., Enghoff H, Epler J, Ewers-Saucedo C, Faber M, Feist S, Figueroa D, Finn J, Fišer C, Fordyce E, Foster W, Frank JH, Fransen C, Furuya H, Galea H, Garcia-Alvarez O, Garic R, Gasca R, Gaviria-Melo S, Gerken S, Gheerardyn H, Gibson D, Gil J, Gittenberger A, Glasby C, Glover A, Gómez-Noguera SE, González-Solís D, Gordon D, Grabowski M, Gravili C, Guerra-García JM, Guidetti R, Guiry MD, Hadfield KA, Hajdu E, Hallermann J, Hayward B, Hendrycks E, Herbert D, Herrera Bachiller A, Ho J s., Høeg J, Hoeksema B, Holovachov O, Hooper J, Houart R, Hughes L, Hyžný M, Iniesta LFM, Iseto T, Ivanenko S, Iwataki M, Jarms G, Jaume D, Jazdzewski K, Kantor Y, Karanovic I, Karthick B, Kim YH, King R, Kirk PM, Klautau M, Kociolek JP, Köhler F, Kolb J, Kotov A, Krapp-Schickel T, Kremenetskaia A, Kristensen R, Kulikovskiy M, Kullander S, Perna R La, Lambert G, Lazarus D, Coze F Le, LeCroy S, Leduc D, Lefkowitz EJ, Lemaitre R, Liu Y, Lörz AN, Lowry J, Ludwig T, Lundholm N, Macpherson E, Madin L, Mah C, Mamos T, Manconi R, Mapstone G, Marek PE, Marshall B, Marshall DJ, Martin P, McInnes S, Meidla T, Meland K, Merrin K, Mesibov R, Messing C, Miljutin D, Mills C, Moestrup Ø, Mokievsky V, Molodtsova T, Monniot F, Mooi R, Morandini AC, Moreira da Rocha R, Moretzsohn F, Mortelmans J, Mortimer J, Musco L, Neubauer TA, Neubert E, Neuhaus B, Ng P, Nguyen AD, Nielsen C, Nishikawa T, Norenburg J, O’Hara T, Okahashi H, Opresko D, Osawa M, Ota Y, Páll-Gergely B, Patterson D, Paxton H, Peña Santiago R, Perrier V, Perrin W, Petrescu I, Picton B, Pilger JF, Pisera A, Polhemus D, Potapova M, Pugh P, Read G, Reimer JD, Reip H, Reuscher M, Reynolds JW, Richling I, Rimet F, Ríos P, Rius M, Rogers C, Rützler K, Rzhavsky A, Sabbe K, Saiz-Salinas J, Sala S, Santos S, Sar E, Sartori AF, Satoh A, Schatz H, Schierwater B, Schmidt-Rhaesa A, Schneider S, Schönberg C, Schuchert P, Senna AR, Serejo C, Shaik S, Shamsi S, Sharma J, Shear WA, Shenkar N, Shinn A, Short M, Sicinski J, Siegel V, Sierwald P, Simmons E, Sinniger F, Sivell D, Sket B, Smit H, Smit N, Smol N, Souza-Filho JF, Spelda J, Sterrer W, Stienen E, Stoev P, Stöhr S, Strand M, Suárez-Morales E, Summers M, Suttle C, Swalla BJ, Taiti S, Tanaka M, Tandberg AH, Tang D, Tasker M, Taylor J, Taylor J, Tchesunov A, Hove H ten, Poorten JJ ter, Thomas J, Thuesen E V, Thurston M, Thuy B, Timi JT, Timm T, Todaro A, Turon X, Tyler S, Uetz P, Utevsky S, Vacelet J, Vachard D, Vader W, Väinölä R, Vijver B Van de, Meij SE van der, Haaren T van, Soest R van, Syoc R Van, Vanreusel A, Venekey V, Vinarski M, Vonk R, Vos C, Walker-Smith G, Walter TC, Watling L, Wayland M, Wesener T, Wetzel C, Whipps C, White K, Williams D, Williams G, Wilson R, Witkowski A, Witkowski J, Wyatt N, Wylezich C, Xu K, Yasuhara M, Zanol J, Zeidler W *.* (2018) World Register of Marine Species (WoRMS). Available from http://www.marinespecies.org at VLIZ. doi:10.14284/170

Kelble CR, Ortner PB, Hitchcock GL, Dagg MJ, Boyer JN (2010) Temporal and spatial variability of mesozooplankton in a shallow sub-tropical bay: influence of top-down control. *Estuaries and Coasts*, **33**, 723–737.

Kemp DW, Thornhill DJ, Rotjan RD. Iglesias-Prieto R, Fitt WK, Schmidt GW (2015) Spatially distinct and regionally endemic *Symbiodinium* assemblages in the threatened Caribbean reef-building coral Orbicella faveolata. *Coral Reefs*, **34**, 535-547.

López-Salgado I, Suárez-Morales E (1998) Copepod assemblages in surface waters of the western Gulf of Mexico. *Crustaceana*, **71**, 312–330.

Montano S, Galli P, Hoeksema BW (2017) First record from the Atlantic: a *Zanclea*-scleractinian association at St. Eustatius, Dutch Caribbean. *Marine Biodiversity*, **47**, 81–82.

Pantos, O, Bythell JC (2010) A novel coral reef symbiosis. *Coral Reefs*, **29**, 761-770.

Skovgaard A, Karpov SA, Guillou L (2012) The parasitic dinoflagellates *Blastodinium* spp. inhabiting the gut of marine, planktonic copepods: morphology, ecology, and unrecognized species diversity. *Frontiers in Microbiology*, **3**, 305.

Stat M, Carter D, Hoegh-Guldberg O (2006) The evolutionary history of *Symbiodinium* and scleractinian hosts—symbiosis, diversity, and the effect of climate change. *Perspectives in Plant Ecology, Evolution and Systematics*, **8**, 23–43.

Strickler KM, Fremier AK, Goldberg CS (2015) Quantifying effects of UV-B, temperature, and pH on eDNA degradation in aquatic microcosms. *Biological Conservation*, **183**, 85.

Thuesen E V, Goetz FE, Haddock SHD (2010) Bioluminescent organs of two deep-sea arrow worms, *Eukrohnia fowleri* and *Caecosagitta macrocephala*, with further observations on bioluminescence in chaetognaths. *The Biological Bulletin*, **219**, 100–111.

Figures:


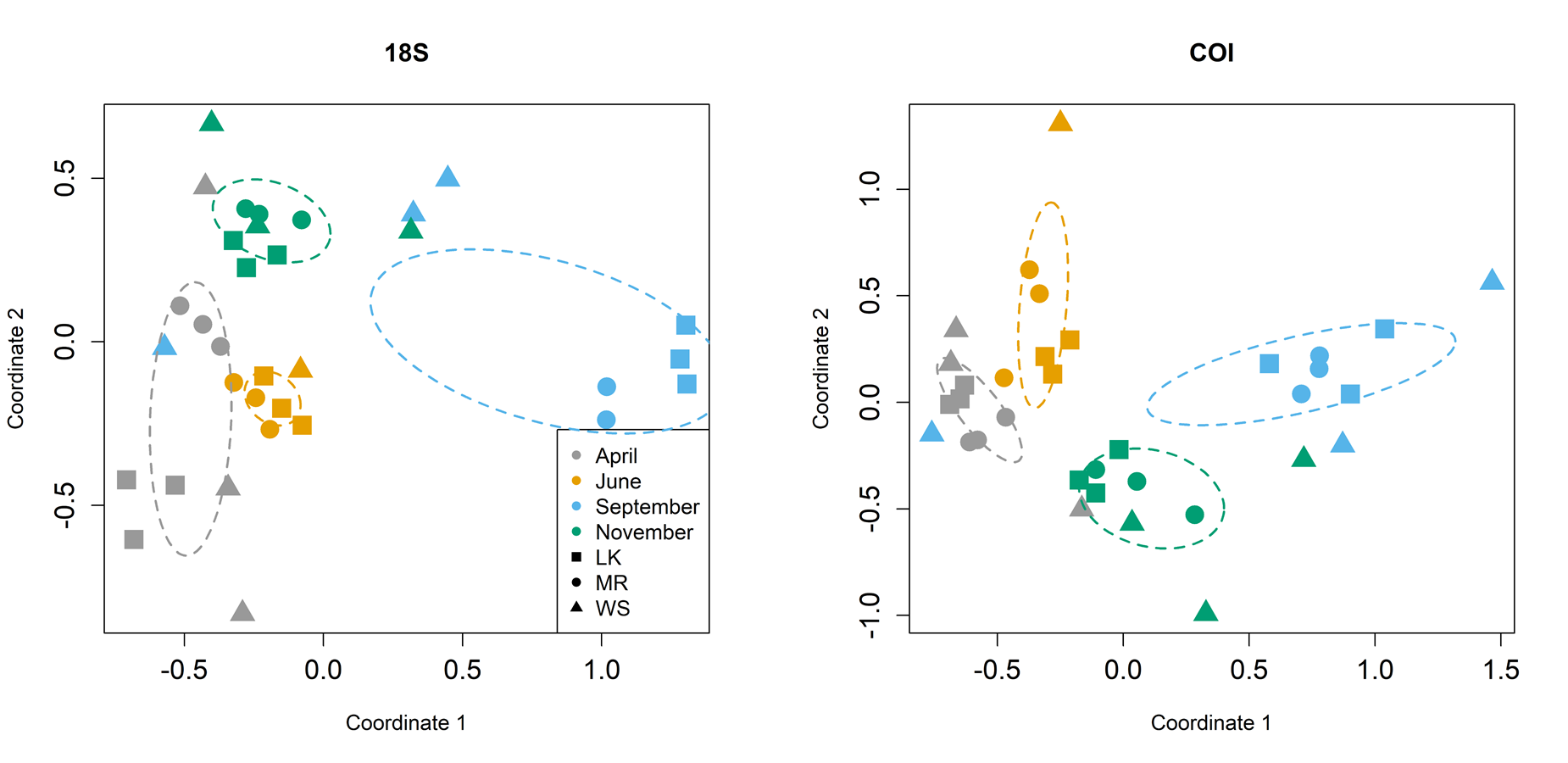


Supporting Figure S1: The NMDS comparison of community structure among samples, with all OTUs found in the filtration control and extraction blank removed. There remains clear clustering, with the months being significantly different from one another (p < 0.05)


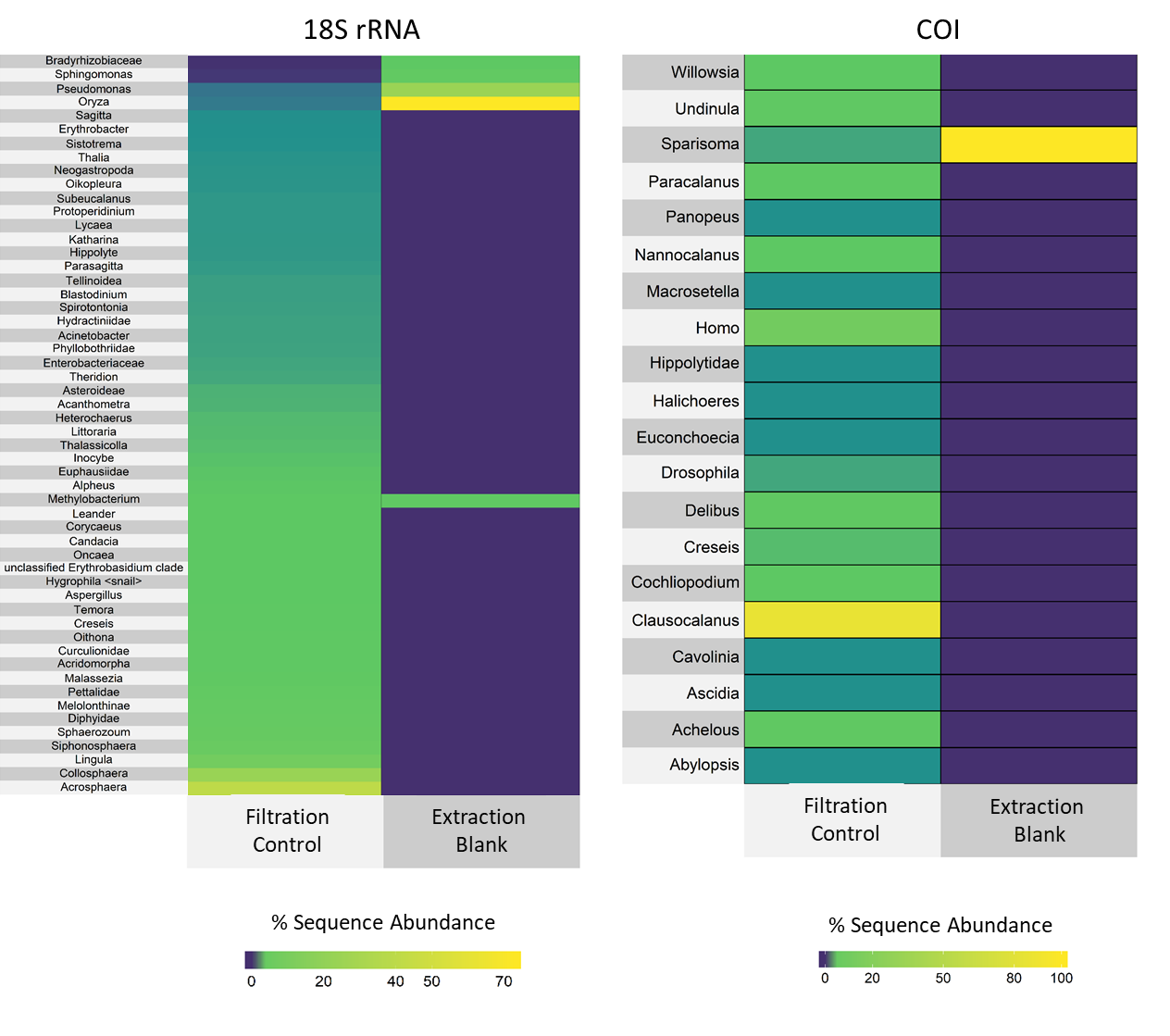


Supporting Figure S2: A heatmap of the percent abundance of the top genera recovered by the filtration and extraction blanks for both 18S rRNA and COI. The 18S rRNA blanks recovered 209 genera so only the top 50, which made up approximately 97% of the annotated reads recovered by the filtration control, are shown. Only 5 genera were recovered in the 18S rRNA extraction blank, four of which were not present in the top 50 of the filtration control and have been added to the heatmap.

Supporting Table S1: A table of all the genera recovered by the 18S filtration control, the percent sequence abundance of all the annotated reads are shown. The top 50 from this table are represented in the heatmap above.

| Genus | Filtration Control |
| --- | --- |
| *Acanthometra* | 0.196905 |
| *Acartia* | 0.008779 |
| *Acetobacteraceae* | 0.017558 |
| *Acinetobacter* | 0.141721 |
| *Acridomorpha* | 0.862869 |
| *Acrochaete* | 0.001254 |
| *Acrosphaera* | 40.23629 |
| *Ala* | 0.065217 |
| *Alexandrium* | 0.001254 |
| *Allium* | 0.020067 |
| *Alopiinae* | 0.037625 |
| *Alpheus* | 0.260867 |
| *Amoebophrya* | 0.001254 |
| *Amphibelone* | 0.012542 |
| *Amphidinium* | 0.001254 |
| *Anteholosticha* | 0.022575 |
| *Apionsoma* | 0.002508 |
| *Aspergillus* | 0.367472 |
| *Asteroidea* | 0.035117 |
| *Asteroideae* | 0.193142 |
| *Aurelia* | 0.036371 |
| *Bathycoccus* | 0.001254 |
| *Batrachochytrium* | 0.006271 |
| *Blastodinium* | 0.131688 |
| *Bradymyces* | 0.010033 |
| *Bradyrhizobiaceae* | 0.001254 |
| *Branchiostoma* | 0.001254 |
| *Calanus* | 0.006271 |
| *Calliacantha* | 0.002508 |
| *Candacia* | 0.334863 |
| *Centropages* | 0.061454 |
| *Cephalothrix* | 0.012542 |
| *Ceratium* | 0.001254 |
| *Cerithioidea* | 0.016304 |
| *Chaetoceros* | 0.008779 |
| *Chrysemys* | 0.005017 |
| *Chrysochromulina* | 0.003763 |
| *Cidaridae* | 0.005017 |
| *Clostridium* | 0.008779 |
| *Coccinellini* | 0.016304 |
| *Cochliopodium* | 0.025083 |
| *Collosphaera* | 23.11937 |
| *Collozoum* | 0.037625 |
| *Corethron* | 0.002508 |
| *Cortinarius* | 0.055183 |
| *Corycaeus* | 0.329847 |
| *Corymorpha* | 0.008779 |
| *Creseis* | 0.548072 |
| *Cryptomonas* | 0.026338 |
| *Cupressaceae* | 0.001254 |
| *Curculionidae* | 0.841548 |
| *Cyclotella* | 0.010033 |
| *Cystobacterineae* | 0.002508 |
| *Dasybranchus* | 0.002508 |
| *Desmophyllum* | 0.018813 |
| *Dinophysis* | 0.005017 |
| *Diogenidae* | 0.002508 |
| *Diphyidae* | 2.710262 |
| *Dipurena* | 0.068979 |
| *Ditrysia* | 0.010033 |
| *Doliolum* | 0.068979 |
| *Duboscquella* | 0.001254 |
| *Echinamoeba* | 0.043896 |
| *Enterobacteriaceae* | 0.153009 |
| *Entomobryidae* | 0.001254 |
| *environmental samples <Eukaryotae>* | 0.005017 |
| *Ephelota* | 0.012542 |
| *Ephydatia* | 0.026338 |
| *Epistylis* | 0.04515 |
| *Erythrobacter* | 0.085284 |
| *Eubranchus* | 0.001254 |
| *Eulalia <polychaete>* | 0.001254 |
| *Euphausiidae* | 0.253342 |
| *Euterpina* | 0.008779 |
| *Eutintinnus* | 0.001254 |
| *Fragilidium* | 0.001254 |
| *Gallus* | 0.010033 |
| *Gemmata* | 0.003763 |
| *Genocidaris* | 0.013796 |
| *Gonyaulax* | 0.036371 |
| *Gymnodinium* | 0.002508 |
| *Gyrodinium* | 0.002508 |
| *Haemophilus* | 0.005017 |
| *Haliangium* | 0.021321 |
| *Haminoeidae* | 0.027592 |
| *Helicostomella* | 0.001254 |
| *Hemiarthrus* | 0.055183 |
| *Hemiophrys* | 0.052675 |
| *Herpotrichiellaceae* | 0.001254 |
| *Heterocapsa* | 0.003763 |
| *Heterochaerus* | 0.216971 |
| *Heterolobosea* | 0.007525 |
| *Heterorhabdidae* | 0.001254 |
| *Hippolyte* | 0.109113 |
| *Hydractiniidae* | 0.140467 |
| *Hygrophila <snail>* | 0.35493 |
| *Inocybe* | 0.242055 |
| *Katharina* | 0.107859 |
| *Kurtiella* | 0.001254 |
| *Laevicardium* | 0.001254 |
| *Latreutes* | 0.035117 |
| *Leander* | 0.328593 |
| *Leptochela* | 0.007525 |
| *Lingula* | 7.724434 |
| *Linuche* | 0.001254 |
| *Liriope* | 0.067725 |
| *Littoraria* | 0.221988 |
| *Lotharella* | 0.001254 |
| *Lycaea* | 0.106604 |
| *Malassezia* | 1.272983 |
| *Melolonthinae* | 2.612436 |
| *Methylobacterium* | 0.27968 |
| *Metridia* | 0.006271 |
| *Metschnikowia* | 0.072742 |
| *Minutocellus* | 0.040133 |
| *Miracia* | 0.001254 |
| *Morella* | 0.005017 |
| *Musculus* | 0.002508 |
| *Naididae* | 0.035117 |
| *Naked dinoflagellate UDNSW0701* | 0.002508 |
| *Nannochloris* | 0.016304 |
| *Navicula* | 0.032608 |
| *Neisseria* | 0.001254 |
| *Neogastropoda* | 0.097825 |
| *Nitrospirae* | 0.002508 |
| *Novosphingobium* | 0.003763 |
| *Ochetostoma* | 0.010033 |
| *Oikopleura* | 0.099079 |
| *Oithona* | 0.782602 |
| *Oncaea* | 0.341134 |
| *Ophiocoma* | 0.01505 |
| *Opitutus* | 0.005017 |
| *Oryza* | 0.063963 |
| *Ostreococcus* | 0.006271 |
| *Owenia* | 0.002508 |
| *Pagrus* | 0.001254 |
| *Palythoa* | 0.031354 |
| *Paracalanus* | 0.001254 |
| *Parasagitta* | 0.116638 |
| *Paulsenella* | 0.001254 |
| *Pectinidae* | 0.001254 |
| *Penicillium* | 0.032608 |
| *Penilia* | 0.048913 |
| *Pettalidae* | 1.749567 |
| *Phascolosoma* | 0.010033 |
| *Phyllobothriidae* | 0.14423 |
| *Phyllodoce <polychaete>* | 0.010033 |
| *Pinnidae* | 0.018813 |
| *Pinus* | 0.001254 |
| *Pirsonia* | 0.001254 |
| *Pleurosigma* | 0.007525 |
| *Polynoidae* | 0.008779 |
| *Portunus* | 0.020067 |
| *Prionospio* | 0.001254 |
| *Prosthecobacter* | 0.003763 |
| *Proterythropsis* | 0.001254 |
| *Protoperidinium* | 0.106604 |
| *Pseudomonas* | 0.058946 |
| *Pseudosolenia* | 0.0301 |
| *Pseudotontonia* | 0.003763 |
| *Pucciniaceae* | 0.056438 |
| *Rhabdonella* | 0.003763 |
| *Rhaphidozoum* | 0.001254 |
| *Rhizosolenia* | 0.073996 |
| *Rhodobacter* | 0.002508 |
| *Rubritalea* | 0.001254 |
| *Saccharomycetaceae* | 0.001254 |
| *Sagitta* | 0.082775 |
| *SAR11 cluster* | 0.003763 |
| *Scambicornus* | 0.001254 |
| *Scrippsiella* | 0.01505 |
| *Sergia* | 0.001254 |
| *Siphonosphaera* | 4.323124 |
| *Sistotrema* | 0.086538 |
| *Smaragdia* | 0.005017 |
| *Spatangoida* | 0.006271 |
| *Sphaerozoum* | 3.284672 |
| *Sphingomonas* | 0.006271 |
| *Spio* | 0.022575 |
| *Spirotontonia* | 0.134196 |
| *Stenostomum* | 0.018813 |
| *Strombidium* | 0.003763 |
| *Subeucalanus* | 0.106604 |
| *Symbiodinium* | 0.026338 |
| *Synaptidae* | 0.031354 |
| *Syndinium* | 0.057692 |
| *Tanytarsus* | 0.002508 |
| *Tellinoidea* | 0.131688 |
| *Temora* | 0.435197 |
| *Teratosphaeria* | 0.041388 |
| *Thalassema* | 0.007525 |
| *Thalassicolla* | 0.229513 |
| *Thalassiosira* | 0.036371 |
| *Thalia* | 0.095317 |
| *Theridion* | 0.161788 |
| *Thoracotremata* | 0.058946 |
| *Trachycaris* | 0.007525 |
| *Truncatelloidea* | 0.01505 |
| *Trypanosoma* | 0.067725 |
| *Typhloscolex* | 0.001254 |
| *unclassified Dothideales* | 0.001254 |
| *unclassified Erythrobasidium clade* | 0.351168 |
| *unclassified Facetotecta* | 0.013796 |
| *unclassified Mycosphaerellaceae* | 0.048913 |
| *unclassified Pleosporaceae* | 0.027592 |
| *Vampyrophrya* | 0.073996 |
| *Vigna* | 0.001254 |
| *Vorticella* | 0.001254 |
| *Zanclea* | 0.001254 |
